# Nitrogen Fertilizer Reduction in Rice–Eel Co-Culture System Improves the Soil Microbial Diversity and Its Functional Stability

**DOI:** 10.3390/plants14152425

**Published:** 2025-08-05

**Authors:** Mengqian Ma, Weiguang Lv, Yu Huang, Juanqin Zhang, Shuangxi Li, Naling Bai, Haiyun Zhang, Xianpu Zhu, Chenglong Xu, Hanlin Zhang

**Affiliations:** 1 Eco-Environmental Protection Institute, Shanghai Academy of Agricultural Science, Shanghai 201403, China; mamengqian191529@163.com (M.M.); lvweiguang@saas.sh.cn (W.L.); bbhy05062023@163.com (Y.H.); zhangjuanqin@saas.sh.cn (J.Z.); lsx1986@126.com (S.L.); bainaling@saas.sh.cn (N.B.); zhanghaiyun.1990@163.com (H.Z.); zhuxp@saas.sh.cn (X.Z.); xuchenglong@saas.sh.cn (C.X.); 2 Key Laboratory of Low-Carbon Green Agriculture in Southeastern China, Ministry of Agriculture and Rural Affairs, Shanghai 201403, China; 3 Key Laboratory of Integrated Rice-Fish Farming Ecosystem, Ministry of Agriculture and Rural Affairs, Shanghai 201403, China; 4 National Agricultural Experimental Station for Agricultural Environment, Fengxian, Shanghai 201403, China

**Keywords:** paddy soil, rice–eel co-culture system, soil bacterial community, earthworms

## Abstract

The ecological rice–eel co-culture system is not only beneficial for enhancing productivity and sustainability in agriculture but also plays a crucial role in promoting environmental health. In the present study, based on the long-term positioning trial of the rice–eel co-culture system that began in 2016 and was sampled in 2023, the effects of reduced nitrogen fertilizer application on soil physico-chemical properties and the bacterial community were investigated. Treatments included a conventional regular fertilization treatment (RT), rice–eel co-culture system regular fertilization (IT), and nitrogen-reduction 10%, 30%, and 50% fertilization treatments (IT90, IT70, and IT50). Our research demonstrated the following: (1) Compared to RT, IT significantly increased soil water-stable macroaggregates (R0.25), mean weight diameter (MWD), geometric mean diameter (GMD), and available phosphorus content, with the increases of 15.66%, 25.49%, 36.00%, and 18.42%, respectively. Among the nitrogen-reduction fertilization treatments, IT90 showed the most significant effect. Compared to IT, IT90 significantly increased R0.25, MWD, GMD, and available nitrogen content, with increases of 4.4%, 7.81%, 8.82%, and 28.89%, respectively. (2) Compared to RT, at the phylum level, the diversity of Chloroflexi was significantly increased under IT and IT50, and the diversity of Gemmatimonadota was significantly increased under IT90, IT70, and IT50. The diversity of Acidobacteriota was significantly higher in IT90 and IT70 compared to IT. It was shown that the rice–eel co-culture system and nitrogen fertilizer reduction could effectively improve the degradation capacity of organic matter and promote soil nitrogen cycling. In addition, redundancy analysis (RDA) identified total phosphorus, total nitrogen, and available nitrogen (*p* = 0.007) as the three most important environmental factors driving changes in the bacterial community. (3) The functional prediction analysis of soil microbiota showed that, compared to RT, the diversity of pathways related to biosynthesis (carbohydrate biosynthesis and cell structure biosynthesis) and metabolism (L-glutamate and L-glutamine biosynthesis) was significantly higher under IT70, IT90, IT, and IT50 (in descending order). However, the diversity of pathways associated with degradation/utilization/assimilation (secondary metabolite degradation and amine and polyamine degradation) was significantly lower under all the rice–eel co-culture treatments. In conclusion, the rice–eel co-culture system improved soil physicochemical properties and the soil microbial environment compared with conventional planting, and the best soil improvement was achieved with 10% less N fertilizer application.

## 1. Introduction

The rice–aquatic animal co-culture system, an integrated farming system combining rice cultivation with fish farming, has a long and rich history, particularly in Asia, where traditions like integrated rice–fish farming have been documented since the Han Dynasty in China (over 2000 years ago). This system fundamentally involves the synergistic cultivation of rice with compatible aquatic species. The core principle lies in creating a mutually beneficial ecosystem. As a highly efficient and sustainable agricultural model, the rice–aquatic animal co-culture system demonstrates significant improvements in economic returns while optimizing land and water resource utilization through complementary resource allocation. This synergistic approach facilitates enhanced land conservation and agricultural productivity, presenting substantial potential for advancing food security, improving water–fertilizer management efficiency, and mitigating agricultural environmental pollution [1,2,3]. The rice–aquatic animal co-culture system includes various combinations of rice–shrimp, rice–fish, rice–crab, rice–turtle, rice–loach, rice–frog, and rice–eel, creating a synergistic material cycle. In this system, aquatic animals such as fish and crabs contribute to the ecosystem by feeding on aquatic plants and small organisms, thereby reducing nutrient loss from the soil and water. A study by Wang et al. [4] found that after 9 years of implementation of the rice–turtle co-culture system, soil available nitrogen (AN), available phosphorus (AP), and organic matter (OM) levels increased by 5.40%, 51.11%, and 23.33%, respectively, compared to monoculture rice fields. The activities of aquatic animals also promote soil aeration, increase soil porosity, and improve the overall soil environment, while predation activities suppress pathogens and pests in rice crops. Studies found that the rice–aquatic animal co-culture system increased total porosity by 6.4% and capillary porosity by 5.7% compared to conventional rice monoculture systems [5]. Pesticide application in the rice–aquatic animal co-culture system can be lowered to 50% of that in high-input rice production [6]. Notably, recent studies have shown that the introduction of earthworms coordinated with the rice–aquatic animal co-culture system can further enhance synergistic effects. As soil ecological engineers, earthworms increase soil porosity through burrowing activities, and their intestinal metabolic processes convert organic matter into humus-rich earthworm manure, which significantly enhances soil cation exchange capacity (CEC) and fast-acting potassium content [7,8]. In the rice–aquatic animal co-culture system, earthworms and aquatic animals form a vertical space complementary—fish stir the surface water to promote oxygen exchange, while earthworms work in the deep soil to build a biological pore network, so that the soil saturated hydraulic conductivity increased by 2–39 times [2,9,10,11].

Meanwhile, the complementary nitrogen utilization between rice and aquatic animals in these systems allows rice to utilize nutrients from animal excreta and uneaten feed, thereby improving the efficiency of nitrogen use in the ecosystem and reducing environmental pollution [12]. Therefore, the rice–aquatic animal co-culture system provides a feasible approach to reduce N fertilizer application [13]. Nitrogen fertilizer application above the optimal level not only adversely affects rice quality and yield [14] but also leads to environmental problems such as water eutrophication and soil degradation [15]. Studies have shown that adjusting the timing and amount of N fertilizer application, particularly during the panicle and tillering stages, can further reduce N fertilizer use by 20% to 50% while maintaining rice yield [11]. Even with a 33.3% reduction in nitrogen fertilizer, rice–crayfish co-culture systems maintain equivalent rice production compared to monoculture systems [11]. This system also increases the effective panicle number of rice and the partial productivity of nitrogen fertilizer. In summary, the rice–aquatic animal co-culture system with reduced nitrogen fertilizer application improves nitrogen availability and cycling, enhances soil fertility, reduces environmental impact, and provides economic benefits. However, few studies have addressed the integrated assessment of yield and long-term soil health changes under these systems.

Soil microbial communities play a pivotal role in nutrient cycling, organic matter decomposition, disease suppression, and soil structure improvement, all of which are essential for ecosystem sustainability [16]. Changes in microbial diversity and community structure are closely linked to soil fertility and serve as bioindicators of soil health. Soil microorganisms are also crucial in driving the biogeochemical cycles of soil elements, particularly the nitrogen cycle; they affect the form of nitrogen in the soil, which in turn affects the utilization of nitrogen by plants. The rice–aquatic animal co-culture system can significantly enhance the activity and diversity of soil microbial communities. The rice–aquatic animal co-culture system significantly increases bacterial network modularity and keystone taxa diversity. The rice–fish co-culture system enhanced paddy soil fertility, bacterial network stability, and keystone taxa diversity, which were predicted to harbor more abundant genomic potentials for nitrogen turnover (e.g., N fixation, denitrification, NO_3_- reduction), and phosphorus mining (e.g., organophosphate mineralization, phosphorus starvation response, and phosphonatases) compared to the rice monoculture [17,18]. Previous studies have found that the combination of the rice–crayfish co-culture system can promote the proliferation of Actinobacteria, Proteobacteria, and Bacillus, increase soil nitrogen levels, and effectively improve soil fertility [17,19]. However, some studies have also shown that rice–aquatic animal co-culture systems have been observed to decrease the diversity and richness of viral, archaeal, and bacterial communities [19,20].

Among the rice–aquatic animal co-culture systems, the rice–eel co-culture system is particularly noteworthy due to the unique burrowing behavior of eels, which create deep and complex burrows (≥50 cm) [20], compared to those of shallow-burrowing species (10–20 cm) such as crabs and shrimp [21]. These burrows improve soil permeability, nitrogen cycling, and rice root development [19]. The organic matter-rich feces excreted by eels in burrows act as a natural fertilizer to improve soil fertility [22]. Additionally, eels control pest populations by feeding on insects and snails, thereby reducing reliance on chemical pesticides [23]. However, few studies have been conducted on soil microorganisms under the rice–eel co-culture system combined with nitrogen fertilization reduction.

Given the advantages of the rice–eel co-culture system and the current need for nitrogen reduction in agriculture, this study aimed to investigate the effects of different nitrogen application rates on soil physico-chemical properties and microbial communities through a long-term rice–eel co-culture system positioning monitoring test. Our objectives were (1) to detect the effects of reduced nitrogen fertilization on environmental physicochemical properties under the rice–eel co-culture system; (2) to explore the effects of reduced nitrogen fertilization on soil bacterial community diversity and structure under the rice–eel co-culture system; (3) to analyze the special microbial communities and ecological functions affected by nitrogen fertilization reduction in the rice–eel co-culture system. This research provides a theoretical basis for the sustainable and integrated development of the rice–eel co-culture system.

## 2. Results

### 2.1. Effects on Soil Physical and Chemical Properties

The distribution of water-stable aggregates in 0–20 cm soil is shown in Table 1. Compared to RT, IT and IT90 significantly increased soil R0.25, MWD, and GMD, demonstrating increases of 15.66, 25.49, and 36.00%; and 20.70, 35.29, and 48.00%, respectively. This was mainly due to the increase in >2 mm and 1.0–2 mm particle size classes and the decrease in <0.053 mm particle size number. Among them, IT90 had the highest R0.25, MWD, and GMD values.

The total nutrient contents in the 0–20 cm soil are presented in Table 2. There were no significant differences in pH among treatments. Similarly, EC values of all the rice–eel co-culture system treatments showed no statistically significant differences from those of RT. Compared to RT, IT90 significantly increased AN, TN, AP, and SOC by 11.02, 52.63, 109.61, and 38.21%, respectively, achieving the highest values among all the treatments. All the nutrient contents of IT were numerically higher than those of RT, increasing AN, AP, AK, TN, TP, TK, and SOC by 6.78, 3.57, 5.49, 18.42, 72.33, 3.59, and 18.91%, respectively. Compared to RT, IT70 significantly increased AN, AP, AK, and SOC but decreased TN and TK. In contrast, IT50 showed significant increases in TN, TP, TK, AN, and AP, with reductions observed in AK and SOC.

### 2.2. Effects on Soil Enzyme Activities

**Figure 1:**
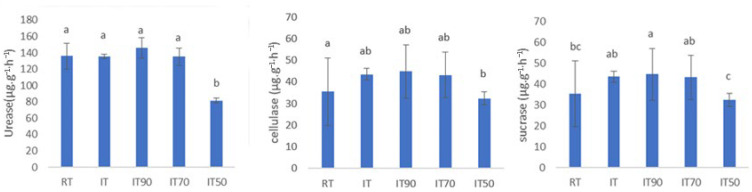
The effects of different fertilization treatments on soil enzyme activity at 0~20 cm. The lowercase letters above the columns denote significant differences between different treatments at *p* < 0.05.

The activities of various soil enzymes under different treatments were assessed, as illustrated in Figure 1. There were no significant differences in the activities of the three enzymes among IT, IT70, and RT. Compared to RT, the sucrase activity of IT90 was significantly increased by 26.34%. The activities of soil urease and cellulase were significantly reduced in the IT50 group compared to RT decreased by 40.18% and 17.05%, respectively.

### 2.3. Effects on Soil Microorganism Gene Copies

**Figure 2:**
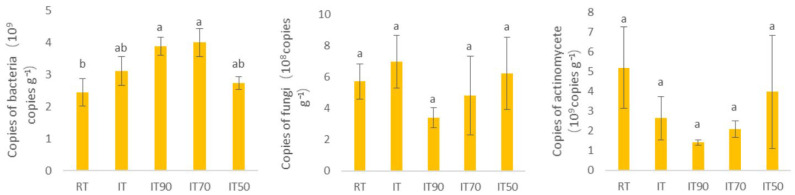
The effects of different fertilization treatments on the biological properties of soil at 0~20 cm. The lowercase letters above the columns denote significant differences between different treatments at *p* < 0.05.

The distribution of microorganism gene copies at 0–20 cm is shown in Figure 2. Each treatment significantly affected bacterial copies. Compared with RT, bacterial copies increased under the rice–eel co-culture system, with IT90 and IT70 showing significantly 58.78% and 63.67% higher values than RT, respectively. In contrast, fungal and actinomycete copies did not differ significantly among all the treatments. However, numerically, actinomycete copies were higher in RT than those in the rice–eel co-culture treatments.

### 2.4. Effects on Bacterial Community Composition

**Figure 3:**
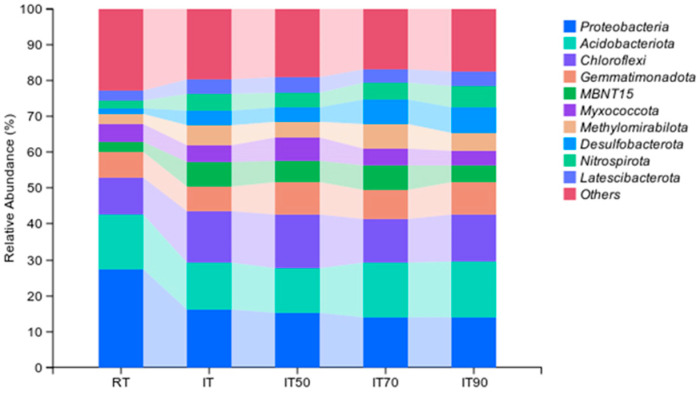
The effects of different fertilization treatments on bacterial community composition at the phylum level. The right side lists different bacterial communities.

As shown in Figure 3 and Table S1, the dominant groups of bacterial community were Proteobacteria, Acidobacteriota, Chloroflexi, and Gemmatimonadota. These four phyla accounted for an average of 44.6% of the community. Compared to RT, the abundance of Chloroflexi increased significantly under IT and IT50, and the abundance of Gemmatimonadota increased significantly under IT90, IT70, and IT50. The abundance of Acidobacteriota was significantly higher under IT90 and IT70 compared to IT. Compared to RT, the abundance of Proteobacteria was significantly reduced in the rice–eel co-culture system.

**Figure 4:**
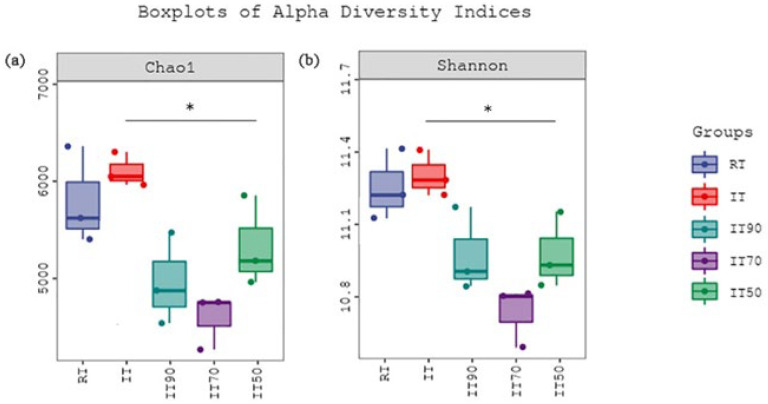
The effects of different fertilization treatments on bacterial α-diversity. Bacterial richness, expressed using the Chao1 index, is shown in (**a**), and diversity, expressed using the Shannon index, is shown in (**b**). Asterisks denote significant differences (*p* * < 0.05).

The effects of different treatments on bacterial α-diversity (*p* < 0.05) are shown in Figure 4. Bacterial richness was expressed using the Chao1 index, and diversity was expressed using the Shannon index. According to Figure 4a, the richness of IT90, IT70, and IT50 was significantly decreased compared to IT, but there was no significant difference between RT and rice–eel co-culture treatments. Figure 4b shows that the bacterial diversity results were similar to the richness results, and the reduction of nitrogen fertilizer in the rice–eel co-culture system resulted in a significant decrease in bacterial diversity.

**Figure 5:**
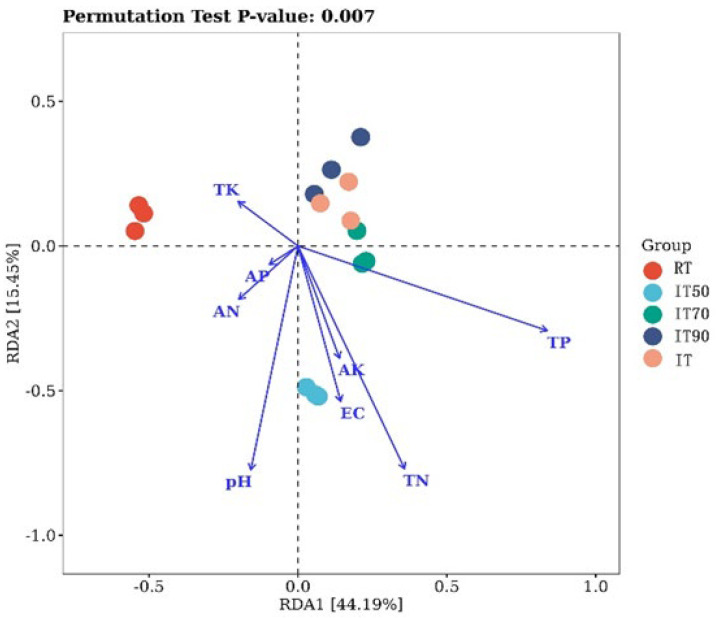
The effects of different fertilization treatments on RDA redundancy analyses. RDA explained 59.6% of the variance in bacterial communities, with the two axes explaining 44.19% and 15.45%, respectively.

Redundancy analysis (RDA) was applied to analyze the correlation between soil physico-chemical properties and bacterial community structure. RDA explained 59.6% of the variance in bacterial communities, with the two axes explaining 44.19% and 15.45%, respectively. In Figure 5, there are significant differences in community composition between traditional and rice–eel co-culture systems, and the two communities were separated by the horizontal axis. AN, TP, and TN (*p* = 0.007) in soil were the three most important factors contributing to changes in the bacterial community. Additionally, IT50 was significantly different from IT90 and IT. TN was the important factor contributing to changes in their bacterial community.

**Figure 6:**
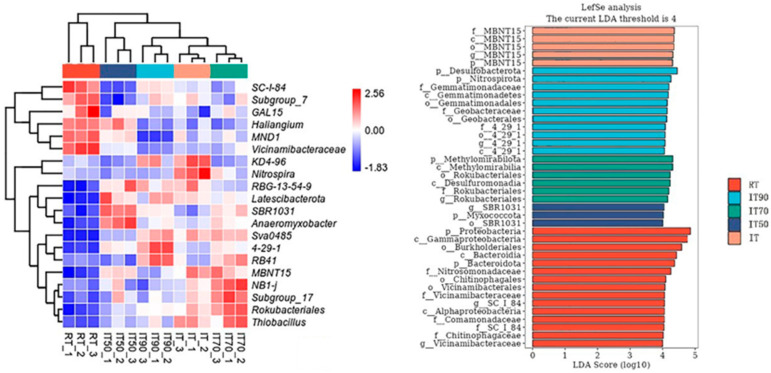
The effects of different fertilization treatments on heat map of bacterial community composition (**left**) and LEfSe analysis (**right**).

Significant differences were found in bacterial community composition at the genus level under the different treatments (Figure 6). SC-1-84 and Vicinamibacteraceae were the dominant genera under RT. Compared with RT, Latescibacterota, Anaeromyxobacter, and SBR1031 were the dominant genera in IT50 soil. RB41, 4-29-1, KD4-96, and Nitrospira were the dominant genera under IT90 and IT, respectively. IT70 increased the abundance of NB1-j, Subgroup_17, Rokubacteriales, and Thiobacillus.

Linear discriminant analysis (LDA) was used to identify differences in bacterial groups among the different treatments. Six bacterial communities at the genus level exhibited linear discriminant analysis (LDA) scores exceeding 4. Among these communities, MBNT15 was notably higher in IT, while 4-29-1, Rokubacteriales, and SBR1031 showed significant variations in IT90, IT70, and IT50, respectively. It is noteworthy that 4-29-1 is the dominant colony at both the phylum and genus levels.

**Figure 7:**
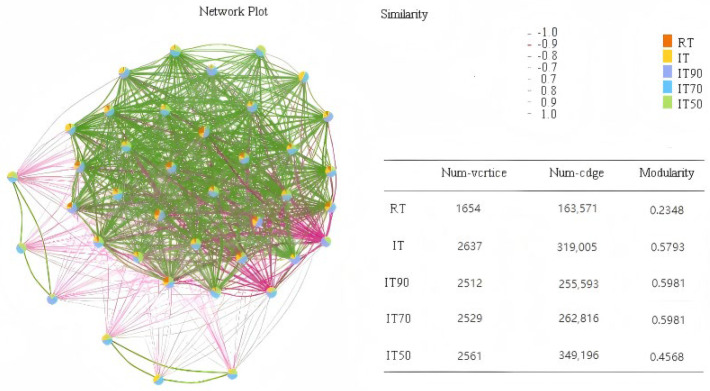
The effects of different fertilization treatments on bacterial community co-occurrence network.

The co-occurrence network and topology of soil bacteria under the different treatments are shown in Figure 7. The results showed that compared to the conventional plantation, the rice–eel co-culture system improved bacterial community stability, which was particularly evident in IT70 and IT90.

### 2.5. Functional Prediction of Soil Microbial Community

**Figure 8:**
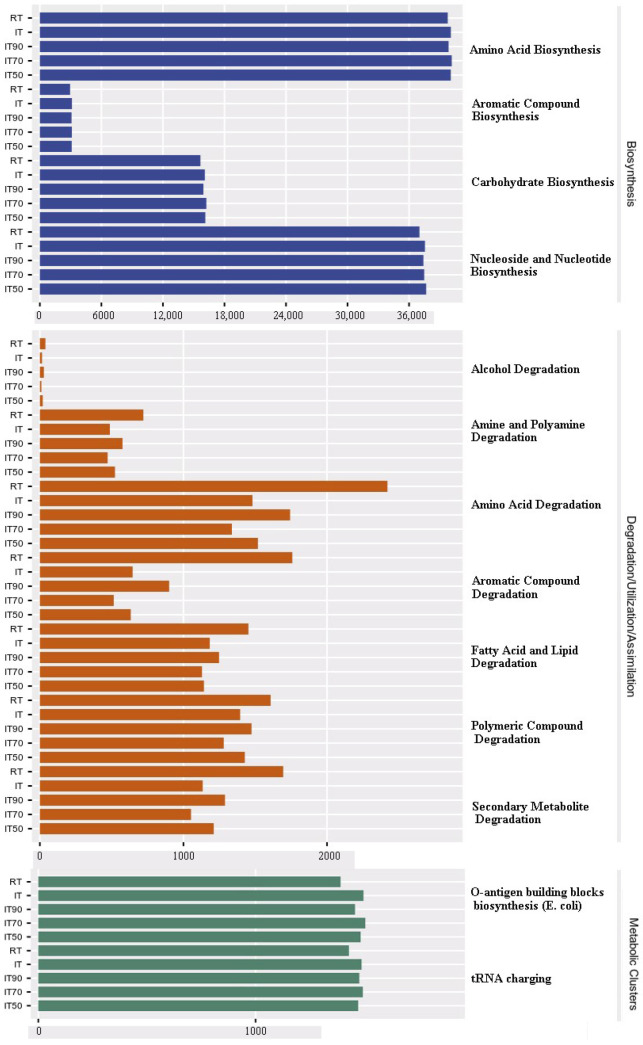
The effects of different fertilization treatments on predicted KEGG pathways in the soil microbial community.

Figure 8 shows the significant differences between KEGG pathways at levels 1 and 2. In comparison with RT, the relative abundance of KEGG pathways related to degradation/utilization/assimilation (e.g., secondary metabolite degradation and amine and polyamine degradation, etc.) was significantly reduced, whereas the relative abundance of biosynthesis (e.g., carbohydrate biosynthesis and aromatic compound biosynthesis, etc.) and metabolism (e.g., biosynthesis of O-antigenic structural units (*E. coli*), etc.) showed a significantly increasing trend in IT70, IT90, IT, and IT50 (in descending order) compared to RT.

## 3. Discussion

### 3.1. The Changes in the Physical and Chemical Properties of Soil

Soil aggregates are fundamental units of soil structure, playing a crucial role in maintaining soil health and functionality. In this study, compared to conventional plantation, it was found that the rice–eel co-culture system increased >2 mm and 1.0–2 mm particle size numbers and decreased <0.053 mm particle size number. This may be due to the eel’s digging activity that creates large holes in the soil, thereby enhancing soil aeration and water infiltration and optimizing soil structure, similar to the findings that soil animals such as earthworms, centipedes, and millipedes improved soil structure by digging holes in soil profiles to form pores that allow soil aeration [24]. In addition, earthworms in paddy fields not only serve as food for eels in the rice–eel system, but the digging behavior of earthworms and eels can also form vertical spatial complements [9]. Eel’s double-vented horizontal burrows (5–8 cm in diameter) were predominantly found in the 10–30 cm soil layer [25], whereas earthworms’ vertical channels (2–5 mm in diameter) ran through the 0–50 cm profile [9,25], and a multi-scale pore network can be formed. R0.25, MWD, and GMD are important indicators of soil aggregate stability, with higher values indicating higher soil structural stability. It has been shown that soil R0.25, MWD, and GMD increased by 9.8%, 13.2% and 30.7% in 15 years in the rice–crayfish system compared to rice monoculture [19]. However, the burrowing ability of eels is much higher than that of other aquatic and soil animals because eels are larger and more active, and may form more extensive and complex burrow systems, and their double-entrance burrow characteristics can alter soil porosity, permeability, stability, and material exchange [26]. In this study, compared with conventional plantation, the R0.25, MWD, and GMD of 10% nitrogen-reducing soils of the rice–eel co-culture system increased by 20.70%, 35.29%, and 48.00%, and the effect of soil structure stabilization was enhanced, which was more significant. It has been shown that excessive nitrogen fertilizer destroys soil pore structure [27], while nitrogen reduction reduces soil sloughing, which, together with the activity perturbation of eels in rice–eel co-cropping (similar to the earthworm effect), significantly increases the proportion of macroaggregates (elevation of MWD and GMD) [28,29]. Therefore, the increase of macroaggregates and the improvement of soil physical properties under reduced nitrogen fertilizer application indicated enhanced soil aggregation and stability [30].

Rice–aquatic animal co-culture systems, such as rice–fish, have been shown to enhance soil quality by increasing soil organic matter content. One meta-analysis found that co-culture systems increased SOC by 11.6% on average compared to rice monoculture systems [31]. In the present study, compared to RT and IT, IT90 soil organic matter increased significantly by 18.89% and 38.16%, respectively, and IT90 had the highest organic matter content, which was in agreement with the results of the previous study. This is due to the additional organic inputs from the aquatic animals’ excreta and the decomposition of organic residues. Meanwhile, moderate nitrogen reduction can reduce the inhibitory effect of chemical nitrogen fertilizers on soil microorganisms, promote the decomposition of rice root secretions and residues, and increase the source of organic matter [32]. The significant decrease in organic matter content of IT70 and IT50 compared to IT90 may be due to the fact that rice growth is impeded when there is a severe nitrogen deficit, resulting in a reduction in biomass (roots, straw) [33], which directly reduces the organic matter input [34]. Microorganisms decompose the original organic matter to obtain nitrogen due to nitrogen scarcity (“nitrogen starvation effect”), accelerating the mineralization and loss of the original organic matter from the soil [35]. The presence of aquatic animals in rice paddies can significantly improve nutrient cycling, particularly phosphorus (P) levels [36]. The effective phosphorus content of the rice–eel co-culture system was significantly increased compared to the monoculture of rice in this study. Integrated farming systems that include aquatic animals like fish and frogs have been shown to increase the concentrations of labile phosphorus fractions in the soil. For instance, the presence of fish in rice paddies significantly increased the concentrations of NaHCO_3_-extractable inorganic phosphorus and organic phosphorus, which are readily available forms of phosphorus for plant uptake [37]. The introduction of eels in this study likewise helped to increase soil reactive phosphorus supply to meet the nutrient requirements of rice with the highest IT50 levels. This is similar to the results of the study in which soluble phosphorus levels were maintained in a rice–fish co-culture system even when fertilizer use was reduced [38,39].

### 3.2. The Changes in Soil Bacterial Diversity and Symbiotic Networks

Soil microorganisms play essential roles in decomposing organic matter, nutrient cycling, and maintaining soil structure [40], which are vital for soil health and fertility [41]. The present study on soil microbial α-diversity found that the rice–eel co-culture system as a whole was not significantly different from conventional plantation, and a previous study found that the effect of the integrated rice–large shrimp co-culture system on soil bacterial community diversity was not significant when compared to conventional plantation [42], which is consistent with the results of the present study. It has been demonstrated that 12 years of the integrated rice–turtle co-culture system significantly altered soil bacterial community diversity, while 7 years of the same practice did not change the diversity of soil bacterial communities [43], so our current 8-year experiment may not have reached the temporal critical threshold for changes in microbial diversity. However, the diversity of IT90, IT70, and IT50 in this study was significantly lower than that of IT, which is consistent with the findings that reduced N fertilizer application leads to lower soil microbial diversity, especially in soils that depend on high N inputs over a long period of time [44,45].

In natural ecosystems, bacteria are not isolated units but are intricately linked, forming complex bacterial communities. Although there was no significant change in microbial diversity between the rice–eel co-culture system and the conventional plantation, in our study, the co-occurrence network analysis showed that the rice–eel co-culture system (especially IT90 and IT70) significantly increased the network complexity and modularity of bacterial communities, implying that the synergistic effect of nitrogen reduction and biotic interactions could enhance the resistance of microbial communities to disturbance, and the advantages of IT90 and IT70 in maintaining higher diversity, marker colony activity, and network stability suggest that they may be the ecologically optimal threshold for nitrogen reduction in the rice–eel co-culture system.

### 3.3. Soil Microbial Community Composition

The bacterial community is a collection of bacterial species and their relative abundance that coexist in a specific environment. Alterations in the specific community may affect ecological functions of the soil, such as nutrient cycling, pathogen suppression, and organic matter decomposition. The dominant bacterial phyla in co-culture systems often include Proteobacteria, Chloroflexi, and Acidobacteria, which are crucial for nutrient cycling and soil health [46]. For example, Lai, Z. et al. found that the rice–shrimp co-culture system changed the composition of the soil microbiota by increasing the relative abundance of certain bacterial phyla (e.g., Acidobacteria and Chloroflexi), while decreasing the relative abundance of other bacterial phyla (e.g., Proteobacteria and Desulfobacteria), which significantly improved the soil fertility [47]. In this study, the bacterial community also changed significantly. The rice–eel co-culture system itself partially compensated for nitrogen loss through eel excreta inputs and maintained bacterial gene copy number and diversity. At the phyla level, the dominant groups are Proteobacteria, Acidobacteriota, Chloroflexi, and Gemmatimonadota. The changes in abundance of Proteobacteria were similar to the results of Lai, Z. et al. studies. The significant decrease in Proteobacteria may be related to their characteristics as eutrophic environment-sensitive taxa, and rice–eel co-culture may have reduced their competitive advantage by altering the carbon and nitrogen cycle. It has been shown that a 20% reduction in nitrogen fertilizer application reduced soil nitrate levels and increased the abundance of functional pathways associated with carbon fixation and nitrogen metabolism, reducing the competitive advantage of Proteobacteria [48]. Meanwhile, Proteobacteria are sensitive to changes in phosphorus availability, and phosphorus addition can alleviate phosphorus deficiency, leading to a decrease in Proteobacteria abundance [49]. The increase in effective phosphorus content of the rice–eel co-culture system in this study may have also led to a decrease in Proteobacteria. The proliferation of Chloroflexi and Gemmatimonadota may have been facilitated by the surge in AP content in the rice–eel co-culture system. Chloroflexi, known for their tolerance to drought and nutrient-poor conditions, showed increased abundance that may be attributed to enhanced organic matter decomposition driven by phosphorus-mediated metabolic activation. The elevated AP likely stimulated phosphatase activity, accelerating organic phosphorus mineralization and providing additional carbon substrates for Chloroflexi. Meanwhile, the rise of Gemmatimonadota, a phylum closely associated with nitrogen cycling, suggesting that the rice–eel co-culture system may have enhanced organic matter decomposition and nitrogen transformation in the soil [50]. Phosphorus availability has been shown to enhance nitrification and denitrification efficiency, potentially creating niche opportunities for Gemmatimonadota to thrive in the restructured nitrogen–phosphorus coupling environment. High abundance of the Acidobacteria phylum in IT90 and IT70 may be associated with increased capacity to utilize complex carbon sources due to increased eel excretion [51]. These interactions ultimately improved nutrient use efficiency and maintained microbial diversity.

### 3.4. The Changes of Soil-Specific Microbial Communities

In addition to the effects of the rice–eel co-culture system, nitrogen fertilizer application influences the composition of bacterial communities because it provides a direct supply of nutrients [52], and this is also key to changes in bacterial communities. Heat map and LEfSe analysis at the genus level showed that nitrogen fertilization reductions drive functional bacterial flora differentiation. In IT50 soil, the emergence of Latescibacterota (related to organic matter degradation [53]), Anaeromyxobacter (parthenogenetic anaerobic bacteria, involved in iron reduction), and SBR1031 (oligotrophic phenotypes) reflected the altered redox state of the soil microenvironment [53,54,55] and increased competition for carbon and nitrogen resources under 50% N reduction. The dominance of Nitrospira under IT may be related to its nitrification function adapted to high-nitrogen environments [56,57]. RB41, a genus of Acidobacteriota that proliferated under 10% nitrogen reduction (IT90), has plant growth-promoting properties such as nitrogen fixation, phosphorus solubilization, and production of phytohormones. When nitrogen supply is appropriately reduced [55], RB41 can enhance its nitrogen acquisition strategy and adapt to low-nitrogen environments [58,59]. However, as nitrogen levels continue to decline, other microorganisms that are more adapted to extremely low nitrogen levels may become dominant, and although RB41 can survive, its competitive disadvantage leads to a decline in abundance. In addition, 4-29-1 (a genus of Acidobacteriota), the dominant bacterial group of IT90, has been shown to prefer soil environments with high organic matter input [60,61], which corresponds to the higher organic matter content of IT90. IT70 exhibited elevated relative abundance of NB1-j (a methane-oxidizing bacteria). Methane-oxidizing bacteria are strict aerobes that rely on O_2_ to oxidize methane (CH_4_) for energy [45,62]. In IT90, high organic matter input may promote the proliferation of heterotrophic microorganisms (such as fermentative bacteria and sulfate-reducing bacteria), which consume large amounts of O_2_ during organic matter decomposition, leading to localized oxygen deficiency in the soil and limiting the survival of methanotrophic bacteria [63]. Additionally, methanotrophic bacteria may be growth-limited due to nitrogen deficiency. This resulted in lower abundance of NB1-j in IT90 and IT50 compared to IT70. These findings suggest that the nitrogen-reduction gradient screens for specific functional taxa adapted to low-nitrogen stress by regulating redox state and resource competition.

### 3.5. Key Environmental Factors Affecting Bacterial Communities

The system modifies key soil environmental factors, which, in turn, influence the composition and interactions of microorganisms. Previous studies have shown that rice–shrimp systems affect bacterial community composition, with the main environmental factors influencing microorganisms being SOM, AK, AP, AN, and pH [64]. The results of our study are similar to those of previous studies. In our study, RDA revealed the dominant role of soil factors in shaping microbial structure. AN, TP, and TN (*p* = 0.007) emerged as the most critical drivers of bacterial community shifts. These nutrients may select for colonies that specialize in nitrogen and phosphorus cycling, mirroring findings in previous studies. At the same time, with the reduction of nitrogen fertilizer, IT, IT90, and IT50 were significantly different from the bacterial community results due to changes in TN. IT50 was significantly different from IT90 and IT. Overall, these findings emphasize that rice–eel co-culture systems enhance soil fertility and microbial diversity through nutrient-mediated shifts in bacterial communities. Optimizing key factors like AN, TP, and TN can promote microbiota optimization, supporting sustainable soil health.

### 3.6. Prediction of Soil Microbial Functionality

The functionality of soil microorganisms refers to the various ecological functions that microorganisms undertake in the soil ecosystem, including decomposition of organic matter, nitrogen fixation, soil nutrient cycling, and pathogen suppression. The potential functions of microorganisms can be predicted by analyzing their genomic information, and KEGG pathway analysis further reveals the functional transition of the system. The reduction of degradation pathways (e.g., secondary metabolite degradation) versus the enhancement of anabolic/metabolic pathways (synthesis of carbohydrates, aroma compounds) in the treatments under the rice–eel co-culture system may reflect that the increase in organic inputs (detritus, feces) promoted microbial anabolic-oriented resource allocation strategies. For example, the enrichment of the Escherichia coli O-antigen synthesis pathway may have resulted from the diffusion of eel gut flora into the soil, whereas elevated carbohydrate synthesis may enhance soil carbon storage potential [65]. However, the reduction of degradation pathways may affect the removal potential of organic pollutants. Therefore, these changes in microbial community functions in the rice–eel co-culture system represent a strategic transition from “decomposition” to “synthesis” of ecological functions. This transition has shown significant advantages in enhancing soil carbon sinks, nutrient efficiency, and emission reduction potential. Some of the data on physical and chemical properties presented previously also confirm this point (Table 2). At the same time, under nitrogen reduction treatment, IT70 had the most significant impact on bacterial function. As shown in Table S2, IT70 had the highest relative abundance in most of the listed biosynthesis and metabolism pathways, while it had the lowest relative abundance in all of the listed degradation/utilization/assimilation pathways. This functional differentiation enables the rice–eel co-culture system to maintain more stable microbial community functions under 30% moderate nitrogen reduction conditions, which is consistent with the results of the microbial symbiotic network analysis in this study. Moderate nitrogen reduction (20–40%) has been shown to enhance microbial interactions and increase the complexity of microbial networks. This reduction does not significantly affect crop yield but improves nitrogen use efficiency and soil health, thereby supporting stable microbial communities [66,67].

## 4. Materials and Methods

### 4.1. Site Description and Experimental Design

The experiment was conducted at the Zhuangxing Experimental Base of the Shanghai Academy of Agricultural Sciences, Fengxian District, Shanghai, China (30°53′ N, 121°23′ E). The site has a subtropical monsoon climate, with an average annual temperature of 12 °C and an annual rainfall of 1166.1 mm. The soil texture is clay loam. The initial soil physicochemical indexes of the experimental plots in 2016 were as follows: pH 7.45; soil organic matter (SOM), 16.43 g·kg^−1^; total nitrogen (TN), 1.05 g·kg^−1^; total phosphorus (TP), 1.14 g·kg^−1^; available nitrogen (AN) 74.47 mg·kg^−1^; and available phosphorus (AP), 34.31 mg·kg^−1^.

**Figure 9:**
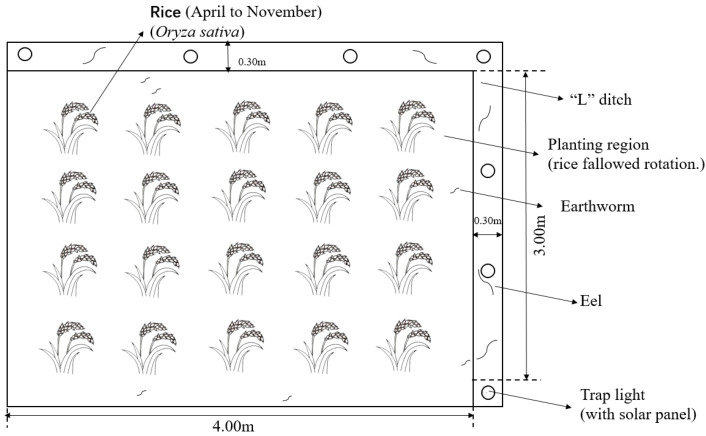
Plan of the experimental plot.

The long-term positioning trial started in the rice season of 2016. The rice variety planted is Hua You 14, and the cultivation pattern is rice–fallow rotation. Five treatments were set up in the trial (Table 3): conventional regular fertilization treatment (RT), co-culture + regular fertilization treatment (IT), co-culture + 10% nitrogen reduction fertilization (IT90), co-culture + 30% nitrogen reduction fertilization (IT70), and co-culture + 50% nitrogen reduction fertilization (IT50). A randomized block design was used, with three replications for each treatment. The plot area for one replication was 12 m^2^, and eels were placed in the “L”-shaped ditch (Figure 9).

### 4.2. Soil Sampling

Soil samples were collected in November 2023, after the rice harvest, when the weather in Shanghai was relatively dry (no rainfall) and the temperature was generally stable (12–18 °C). The top-layer soil samples (0–20 cm) were randomly collected with a soil sampler from 10 points in each replicate and thoroughly mixed into a single sample. The soil samples were stored in sterilized, sealed polyethylene bags and immediately transported to the laboratory. One portion of the samples was freeze-dried for soil physical and chemical property measurements, and the other portion was stored at −80 °C for DNA extraction and high-throughput sequencing analysis.

### 4.3. Indicator Measurement and Methodology

#### 4.3.1. Determination of Soil Aggregates

The water-stable agglomerates were graded by the wet sieve method, and six classes of water-stable agglomerates—>2 mm, 1~2 mm, 0.5~1 mm, 0.25~0.5 mm, 0.053~0.25 mm, and <0.053 mm—were separated using the DIK-2012 soil agglomerates analyzer and were weighed after drying them on an electric hot plate. The water-stable macroaggregates (R0.25), mean weight diameter (MWD), and geometric mean diameter (GMD) were calculated as follows:

(1) $$R0.25=\frac{M_{r}>0.25}{MT}$$

(2) $$MWD=\sum_{i=1}^{n}W_{i}X_{i}$$

(3) $$GMD=\exp\left(\frac{\sum_{i=1}^{n}W_{i}lnX_{i}}{\sum_{i=1}^{n}W_{i}}\right)$$

where M_r_ > 0.25 is the weight of agglomerates with particle size >0.25 mm, g; MT is the total weight of agglomerates, g; R0.25 is the proportion of water-stable agglomerates, %; X_i_ is the average diameter of agglomerates of particle size i, mm; W_i_ is the percentage of agglomerates of particle size corresponding to X_i_ relative to the total weight of the soil, %; MWD is the mean weight diameter, mm; and GMD is the geometric mean diameter, mm.

#### 4.3.2. Determination of Soil Nutrients and Enzyme Activity

Soil pH was measured with a pH meter (InPro2000, Mettler-Toledo, Switzerland) at a soil: water ratio of 1:2.5.; soil TN and AN were determined by the Kjeldahl method (extracted by 2 M KCl); soil TP was determined using the molybdenum-blue colorimetry method (dissolved by 5 M H_2_SO_4_), and AP was determined using the molybdenum blue method (extracted by 0.5 M NaHCO3); soil total potassium (TK) and available potassium (AK) were determined with flame photometry (FP-6410; Xinyi Instruments., Shanghai, China); and SOM was determined using the potassium dichromate method (treated with K_2_Cr_2_O_7_ solution mixed with H_2_SO_4_).

Determination of soil urease (S-UE): The activity of urease was indicated using the indophenol blue colorimetric method. Determination of soil sucrase (S-SC): The activity of sucrase was indicated using the 3,5-dinitrosalicylic acid method. Determination of soil cellulase (S-CL): The activity of cellulase was indicated using the 3,5-dinitrosalicylic acid method.

#### 4.3.3. DNA Extraction and High-Throughput Sequencing

Soil DNA was extracted using the Mega E.Z.N.A.^®^ Soil DNA Kit (D5625-02, Omega Bio-Tek, Norcross, GA, USA) according to the manufacturer’s instructions. The number of soil bacteria, fungi, and actinomycetes was determined by fluorescence quantitative PCR. The primer information used in the experiment is shown in Table 4.

The PCR amplifications were performed using an Applied Biosystems Real-Time PCR instrument (Applied Biosystems Inc., StepOnePlus, Foster City, CA, USA) under the following cycling conditions: for the 16S V4–V5 rRNA genes, 30 s of denaturation at 98 °C, followed by 25 cycles of 98 °C for 15 s, 50 °C for 30 s, and 72 °C for 30 s, and an extension at 72 °C for 7 min. The PCR products were checked with a NanoDrop ND-1000 UV–Vis spectrophotometer (Thermo Fisher Scientific, NanoDrop 1000, Waltham, MA, USA). Sequencing was performed on an Illumina MiSeq platform (USA) at Personal Biotechnology Co., Ltd. (Shanghai, China). Raw sequences underwent quality control and assigned to unique 10-bp barcodes using QIIME software (version 1.7.0) [26]. Then, the remaining sequences were clustered into operational taxonomic units (OTUs) based on 97% sequence similarity.

### 4.4. Statistical Analyses

Significant differences between treatments were analyzed using a one-way analysis of variance (ANOVA) followed by the Tukey comparison test (*p* < 0.05). Statistical analysis was performed using IBM SPSS Statistics 25, and charting was conducted using Origin 9.0. The bacterial richness, diversity, and evenness indices (Chao1, Shannon, and Simpson) were calculated using Mothur (version v.1.30.1). The species contributing to significant differences in the bacterial and fungal communities were identified using linear discriminant analysis (LDA) effect size (LEfSe). Redundancy analysis (RDA) was used to examine the relationship between soil bacterial communities and environmental factors.

## 5. Conclusions

This study showed that the rice–eel co-culture system can improve soil physical properties and nutrient content through the synergistic effects of eel activity and nitrogen-reducing fertilization, thereby significantly optimizing soil structure, enhancing nutrient effectiveness, and promoting microbial community succession toward carbon–nitrogen cycling. Among the treatments, 10% nitrogen reduction (IT90) was most effective in improving soil physical properties, microbial diversity, and functional stability, providing both theoretical and practical bases for the green and intensive management of paddy ecosystems.

## Figures and Tables

**Table 1:** The effects of different fertilization regimes on soil physical properties.

							R0.25	MWD	GMD
Treatments	Mass Percentage of Water-Stable Aggregates of Different Sizes (%)						(%)	(mm)	(mm)
	>2 mm	1.0~2.0 mm	0.5~1.0 mm	0.25~0.5 mm	0.053~0.25 mm	<0.053 mm			
RT	8.47 ± 0.01 b	7.64 ± 0.01 c	10.85 ± 0.04 b	27.38 ± 0.05 ab	16.45 ± 0.03 ab	29.21 ± 0.06 a	54.34 ± 0.09 b	0.51 ± 0.06 c	0.25 ± 0.05 b
IT	12.57 ± 0.02 a	11.01 ± 0.02 b	11.03 ± 0.02 b	28.24 ± 0.01 a	18.31 ± 0.02 a	18.85 ± 0.03 c	62.85 ± 0.02 a	0.64 ± 0.05 ab	0.34 ± 0.03 a
IT90	12.09 ± 0.01 a	13.55 ± 0.01 a	18.50 ± 0.03 a	21.46 ± 0.02 bc	12.55 ± 0.01 b	21.86 ± 0.02 bc	65.59 ± 0.02 a	0.69 ± 0.03 a	0.37 ± 0.07 a
IT70	7.31 ± 0.01 b	11.19 ± 0.02 ab	13.34 ± 0.03 b	19.55 ± 0.02 c	20.67 ± 0.01 a	27.95 ± 0.06 ab	51.38 ± 0.03 b	0.53 ± 0.06 bc	0.26 ± 0.04 b
IT50	6.25 ± 0.03 b	7.42 ± 0.02 c	18.15 ± 0.03 a	19.08 ± 0.06 c	20.35 ± 0.02 a	28.75 ± 0.03 a	50.89 ± 0.01 b	0.49 ± 0.07 c	0.25 ± 0.03 b

Note: The significant differences are represented by different lowercase letters (*p* < 0.05). R0.25, the water-stable macroaggregates; MWD, mean weight diameter; GMD, geometric mean diameter.

**Table 2:** The effects of different fertilization regimes on soil nutrients.

Treatment	TN	TP	TK	AN	AP	AK	SOC	pH	EC
	(g kg^−1^)	(g kg^−1^)	(g kg^−1^)	(mg kg^−1^)	(mg kg^−1^)	(mg kg^−1^)	(g kg^−1^)		(ms cm^−1^)
RT	1.18 ± 0.09 b	0.56 ± 0.05 b	18.58 ± 2.62 a	31.16 ± 5.74 b	11.13 ± 2.84 b	120.67 ± 10.33 a	11.66 ± 2.06 b	7.81 ± 0.14 a	0.22 ± 0.03 ab
IT	1.26 ± 0.03 ab	0.58 ± 0.02 b	19.60 ± 0.65 a	36.90 ± 9.84 b	19.18 ± 3.63 a	125.00 ± 16.00 a	13.86 ± 1.73 ab	7.60 ± 0.31 a	0.18 ± 0.05 b
IT90	1.31 ± 0.04 a	0.61 ± 0.01 ab	19.40 ± 0.75 a	47.56 ± 4.10 a	23.33 ± 2.42 a	102.33 ± 20.67 ab	16.11 ± 3.98 a	7.79 ± 0.27 a	0.26 ± 0.04 a
IT70	1.17 ± 0.06 b	0.56 ± 0.04 b	15.91 ± 4.46 a	40.18 ± 6.56 ab	21.75 ± 4.84 a	126.00 ± 6.00 a	11.74 ± 0.65 b	7.66 ± 0.27 a	0.21 ± 0.01 ab
IT50	1.19 ± 0.08 b	0.66 ± 0.06 a	19.21 ± 1.19 a	32.80 ± 1.64 b	24.38 ± 5.05 a	87.00 ± 14.00 b	11.20 ± 1.48 b	7.79 ± 0.18 a	0.18 ± 0.04 b

Note: Significant differences are represented by different lowercase letters (*p* < 0.05). TN, soil total nitrogen; TP, soil total phosphorus; TK, soil total potassium; AN, soil available nitrogen; AP, soil available phosphorus; AK, soil available potassium; SOC, soil organic carbon; EC, electrical conductivity.

**Table 3:** Fertilization application of different fertilization regimes.

Treatments	Whether to Breed Eel	Type and Dosage of Fertilizers
RT	No	Regular fertilization
IT	Yes	Regular fertilization
IT90	Yes	10% nitrogen reduction fertilization
IT70	Yes	30% nitrogen reduction fertilization
IT50	Yes	50% nitrogen reduction fertilization

Regular fertilization in this area: Urea 489 kg·hm^−2^, Ca(H_2_PO_4_)_2_ 938 kg·hm^−2^, K_2_SO_4_ 750 kg·hm^−2^.

**Table 4:** Primer information.

Measurement Target	Target Gene	Gene Name	Primer Sequence
bacteria	341F-806R	341F	CCTAYGGGRBGCASCAG
		806R	GGACTACNNGGGTATCTAAT
fungi	ITS1F-ITS2R	ITS1F	CTTGGTCATTTAGAGGAAGTAA
		ITS2R	GCTGCGTTCTTCATCGATGC
actinomycetes	Act920F-Act1200R	Act920F	TACGGCCGCAAGGCTA
		Act1200R	TCRTCCCCACCTTCCTCCG

## Data Availability

All data necessary to replicate this study’s results are included in this published article. Raw data are available upon request. The datasets presented in this study are available in the NCBI repository under accession number PRJNA1262102 (http://www.ncbi.nlm.nih.gov/bioproject/1262102) URL (accessed on 13 May 2025).

## Supplementary Materials

The following supporting information can be downloaded at: https://www.mdpi.com/article/10.3390/plants14152425/s1, Table S1: Bacterial community composition at the phylum level; Table S2: Predicted KEGG pathways in the soil microbial community under different treatments.

## Abbreviations

The following abbreviations are used in this manuscript:

RT	Conventional regular fertilization treatment
IT	Co-culture + regular fertilization treatment
IT90	Co-culture + 10% nitrogen reduction fertilization
IT70	Co-culture + 30% nitrogen reduction fertilization
IT50	Co-culture + 50% nitrogen reduction fertilization
R0.25	Water-stable macroaggregate
MWD	Mean weight diameter
GMD	Geometric mean diameter
TN	Soil total nitrogen
TP	Soil total phosphorus
TK	Soil total potassium
AN	Soil available nitrogen
AP	Soil available phosphorus
AK	Soil available potassium
SOM	Soil organic matter
SOC	Soil organic carbon
EC	Electrical conductivity
RDA	Redundancy analysis
LDA	Linear discriminant analysis
