# Supplementary material for: Nitrogen Fertilizer Reduction in Rice–Eel Co-Culture System Improves the Soil Microbial Diversity and Its Functional Stability
Source: Plants (Basel). 2025 Aug 5;14(15):2425. doi: 10.3390/plants14152425 (PMC12349682; doi:10.3390/plants14152425)
Supplement: Supplementary file 1 [file plants-14-02425-s001.zip › plants-3725638-supplementary.pdf]

Supplementary Materials

Table S1. Bacterial community composition at the phylum level.

KEGG_ level 1	KEGG_ level 2	Relative abundance				
		RT	IT	IT90	IT70	IT50
Biosynthesis	Amino Acid Biosynthesis	c	ab	ab	a	b
	Aromatic Compound Biosynthesis	b	a	a	a	a
	Carbohydrate Biosynthesis	c	ab	a	a	b
	Nucleoside and Nucleotide Biosynthesis	b	a	a	a	a
Degradation/Utilization/Assimilation	Alcohol Degradation	a	c	bc	c	b
	Amine and Polyamine Degradation	a	c	bc	c	b
	Amino Acid Degradation	a	c	c	c	b
	Aromatic Compound Degradation	a	c	c	c	b
	Fatty Acid and Lipid Degradation	a	b	b	b	b
	Polymeric Compound Degradation	a	b	b	c	b
	Secondary Metabolite Degradation	a	cd	bc	d	b
Metabolic Clusters	O-antigen building blocks biosynthesis (E. coli)	c	c	bc	a	b
	tRNA charging	c	ab	b	a	ab

Table S2. Predicted KEGG pathways in the soil microbial community under different treatments.

	RT	IT	IT90	IT70	IT50
Proteobacteria	a	b	b	b	b
Acidobacteriota	a	b	a	a	b
Chloroflexi	b	a	ab	ab	a
Gemmatimonadota	b	b	a	a	a
MBNT15	c	a	b	a	a
Myxococcota	b	b	c	bc	a
Methylomirabilota	d	b	bc	a	c
Desulfobacterota	c	b	a	a	b
Nitrospirota	d	b	a	b	c
Latescibacterota	b	a	a	a	a
